# Shift in Bacterial Community Structure in the Biodegradation of Benzene and Toluene under Sulfate-Reducing Condition

**DOI:** 10.3390/toxics12060423

**Published:** 2024-06-10

**Authors:** Zhengwei Liu, Xiaoyu Lin, Xinzhe Wang, Mingbo Sun, Shici Ma, Shucai Zhang

**Affiliations:** 1 State Key Laboratory of Chemical Safety, Qingdao 266071, China; liuzw.qday@sinopec.com (Z.L.); linxy.qday@sinopec.com (X.L.); wangxz.qday@sinopec.com (X.W.); sunmb.qday@sinopec.com (M.S.); masc.qday@sinopec.com (S.M.); 2 SINOPEC Research Institute of Safety Engineering Co., Ltd., Qingdao 266000, China

**Keywords:** benzene, toluene, sulfate reduction, biostimulation, bacterial community

## Abstract

Groundwater contaminated by benzene and toluene is a common issue, posing a threat to the ecosystems and human health. The removal of benzene and toluene under sulfate-reducing condition is well known, but how the bacterial community shifts during this process remains unclear. This study aims to evaluate the shift in bacterial community structure during the biodegradation of benzene and toluene under sulfate-reducing condition. In this study, groundwater contaminated with benzene and toluene were collected from the field and used to construct three artificial samples: Control (benzene 50 mg/L, toluene 1.24 mg/L, sulfate 470 mg/L, and HgCl_2_ 250 mg/L), S1 (benzene 50 mg/L, toluene 1.24 mg/L, sulfate 470 mg/L), and S2 (benzene 100 mg/L, toluene 2.5 mg/L, sulfate 940 mg/L). The contaminants (benzene and toluene), geochemical parameters (sulfate, ORP, and pH), and bacterial community structure in the artificial samples were monitored over time. By the end of this study (day 90), approximately 99% of benzene and 96% of toluene could be eliminated in both S1 and S2 artificial samples, while in the Control artificial sample the contaminant levels remained unchanged due to microbial inactivation. The richness of bacterial communities initially decreased but subsequently increased over time in both S1 and S2 artificial samples. Under sulfate-reducing condition, key players in benzene and toluene degradation were identified as *Pseudomonas*, *Janthinobacterium*, *Novosphingobium*, *Staphylococcus*, and *Bradyrhizobium*. The results could provide scientific basis for remediation and risk management strategies at the benzene and toluene contaminated sites.

## 1. Introduction

Groundwater contamination by petroleum hydrocarbons has emerged as a pressing environmental issue, largely stemming from oil spills and leaks from underground pipes and storage tanks during oil production, transportation, and storage at various industrial sites [1,2]. Among the suite of petroleum hydrocarbons, BTEX (benzene, toluene, ethylbenzene, and xylene) are of particular concern due to their toxicity and carcinogenicity [3,4]. Moreover, given their relatively high water solubility, BTEX compounds can migrate through natural groundwater flow systems, potentially contaminating distant drinking water sources [5].

In natural environments, microorganisms harness energy for cellular growth and maintenance by facilitating the transfer of electrons from electron donors to electron acceptors [6]. Typically, electron acceptors are elements or compounds existing in relatively oxidized states, and they mainly encompass dissolved oxygen (DO), nitrate (NO_3_^−^), manganese (IV) (Mn^4+^), ferrous iron (Fe^3+^), sulfate (SO_4_^2−^), and carbon dioxide (CO_2_) [6,7,8]. Under most circumstances, biodegradation in aquifers primarily occurs through sulfate reduction under natural conditions. Thierrin et al. have documented BTEX biodegradation under sulfate-reducing condition resulting from a gasoline spill [9]. Thus, the potential utilization of sulfate-reducing processes for the remediation of BTEX-contaminated groundwater presents a practical and cost-effective remedial option for addressing petroleum hydrocarbon contamination [4,10,11,12,13,14,15]. Several studies have detailed the contaminant removal performance achieved through sulfate-reducing processes. For example, Chen et al. report the highest biodegradation rates under sulfate-reducing conditions compared to denitrifying, manganese-reducing, and iron-reducing conditions [4]. This superior performance is attributed to the greater abundance of SO_4_^2−^-reducing bacteria in the culture relative to other reducing bacteria. Lovley et al. demonstrate that under sulfate-reducing conditions, benzene is readily mineralized to CO_2_ and water, without producing intermediate products like phenol, benzoate, or acetate [11]. Huang et al. explore the use of sulfate reduction mechanisms for simultaneous bioremediation of toluene and copper-contaminated groundwater, achieving nearly 99% removal of both contaminants over a 40-day period. The bacterial composition on day 30 included *Citrobacter*, *Klebsiella*, *Acinetobacter*, *Pseudomonas*, *Bacteroides*, *Clostridium*, *Bacteroides*, *Rhodoplanes*, *Betaproteobacteria*, *Zoogloea resiniphila*, and *Dysgonomonas* [14]. Juliana documents that combined biostimulation of iron and sulfate reduction accelerates BTEX and PAH biodegradation in diesel/biodiesel blends, maintaining low dissolved concentrations of benzene and naphthalene throughout the experiment compared to a baseline control under monitored natural attenuation. *Geobacter* spp. and *GOUTA19* spp. appear to play key roles in the anaerobic biodegradation of diesel/biodiesel blends under iron and sulfate reduction [16]. Norma compares the performance of two permeable reactive barriers with differing internal substrate configurations—one containing a sulfate solution without metals, the other with metals—for treating groundwater contaminated with acid mine drainage. Bacterial diversity was higher at the beginning and middle of the experiment in both systems [17].

However, the specific changes in the bacterial community in response to contaminant concentration and reactive phase during the biodegradation of benzene and toluene under sulfate-reducing condition remain unclear. To address this gap, actual benzene and toluene-contaminated groundwater from a petrochemical site was collected to construct artificial samples. Supplementary electron acceptor SO_4_^2−^ was added to investigate the feasibility of enhanced sulfate reduction for remediating benzene and toluene-contaminated groundwater. The study aimed to evaluate the shift in the bacterial community structure during the biodegradation of benzene and toluene under sulfate-reducing condition and identify the key bacteria that are most effective in the biodegradation process of benzene and toluene under sulfate-reducing condition. Understanding the dynamics of microbial communities offers a scientific foundation for optimizing bioremediation strategies. This knowledge enables the enhancement of their activities or the targeted introduction of these microorganisms into environments where they can exert maximum efficacy, thereby improving remediation outcomes.

## 2. Materials and Methods

### 2.1. Groundwater Sampling and Analysis

**Figure 1:**
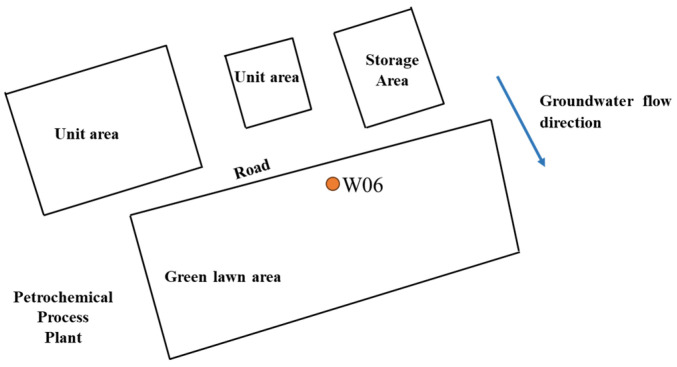
Locations of the groundwater sampling point.

Groundwater contaminated with benzene and toluene was obtained for this study. The research site is situated within a petrochemical complex in southeaster China that has been operational for more than three decades. Figure 1 illustrates the layout of the contaminated site along with the positioning of the well. The aquifer medium primarily consists of sandy clay in the examined depth range. Predominantly, the groundwater type found here is Quaternary phreatic groundwater, which extends from a depth of 3 m to 15 m beneath the surface. The natural flow direction of the groundwater flows predominantly from the northwest towards the southeast. Groundwater samples (W06) were collected using MicroPurge Low flow groundwater sampling system (SamplePRO, QED Environmental Systems Limited Inc., Hong Kong, China) after the construction of the wells.

pH, ORP, and DO were measured in situ using respective portable testers. Ion Chromatography (DIONEXTM AQUIONTM Thermo Scientific, Waltham, MA, USA) was employed to determine SO_4_^2−^ and NO_3_^−^ anions. Cations, particularly Fe^2+^ and Mn^2+^, were quantified using a portable HACH DR3900 analyzer following the standard methods 8146 (1,10-phenanthroline photometric method) and 8034 (periodate method), respectively. The concentrations of benzene and toluene were analyzed using purge-trap and gas chromatography-mass spectrometry (GC-MS, Agilent 7890B 5977B-Atomx XYZ Analytical Instruments, Agilent, Santa Clara, CA, USA), following the procedures outlined in US EPA Method 502.2 [18].

### 2.2. Artificial Sample Setup

Three artificial samples, Control, S1, and S2 have been set up, and their components were shown in Table 1. In the control artificial sample, 250 mg/L HgCl_2_ was added to inhibit microbial activity. The anaerobic artificial samples were assembled in 50 mL glass serum bottles, each filled to capacity with the contaminated groundwater (with benzene and toluene) and sulfate. The contaminant concentrations were adjusted by diluting the groundwater with deionized water to achieve the targeted concentrations. The artificial samples were prepared inside an N_2_ glovebox. The artificial sample experiment was conducted over a 90-day period. On day 1, 3, 20, 40, 60, and 90, samples were collected for analysis of benzene, toluene, sulfate, ORP, and pH. On day 3, 40, and 90, samples were collected for analysis of bacterial community structure.

### 2.3. Analytical Method

At each sampling time point, three artificial sample bottles from each group were analyzed. pH and ORP were measured in situ using respective portable testers. Ion Chromatography (DIONEXTM AQUIONTM Thermo Scientific) was employed to determine SO_4_^2−^ anions. The concentrations of benzene and toluene were analyzed using purge-trap and gas chromatography-mass spectrometry (GC-MS, Agilent 7890B 5977B-Atomx XYZ Analytical Instruments, Agilent, USA), following the procedures outlined in US EPA Method 502.2 [18].

### 2.4. Analysis of Bacterial Community Sturcture

Bacterial DNA was extracted using the MagPure Soil DNA LQ Kit (Magen Biotechnology, Guangdong, Guangzhou, China) following manufacturer’s instructions to investigate the microbial diversity of the contaminated groundwater as shown in Table S1. The integrity and concentration of the extracted DNA were determined using a NanoDrop 2000 spectrophotometer (Thermo Fisher Scientific, Waltham, MA, USA) and agarose gel electrophoresis, respectively. PCR amplification of the V3-V4 hypervariable regions of the bacterial 16S rRNA gene (94 °C for 5 min, followed by 26 cycles at 94 °C for 30 s, 56 °C for 30 s, and 72 °C for 20 s and a final extension at 72 °C for 5 min) was carried out in a 25 μL reaction using universal primer pairs 343F (5′-TACGGRAGGCAGCAG-3′) and 798R (5′-AGGGTATCTAATCCT-3′) [19]. The reverse primer contained a sample barcode and both primers were connected with an Illumina sequencing adapter. The polymerase chain reaction (PCR) reaction system and reaction conditions are shown in Table S1 in the supplemental materials. 

The Amplicon quality was visualized using gel electrophoresis. The PCR products were purified with Agencourt AMPure XP beads (Beckman Coulter Co., Brea, CA, USA) and quantified using Qubit dsDNA assay kit. The concentrations were then adjusted for sequencing. Sequencing was performed on an Illumina NovaSeq6000 with two paired-end read cycles of 250 bases each. (Illumina Inc., San Diego, CA, USA).

Paired-end reads were preprocessed using Trimmomatic software 0.39 to detect and cut off ambiguous bases. It also cut off low quality sequences with average quality score below 20 using sliding window trimming approach. After trimming, paired-end reads were assembled and employed the base with a higher quality score as the output using FLASH software 32.0.0.465. Parameters of assembly were: 10 bp of minimal overlapping, 200 bp of maximum overlapping and 20% of maximum mismatch rate. Sequences were performed further denoising as follows: reads with ambiguous, homologous sequences or below 200 bp were abandoned. Reads with 75% of bases above Q20 were retained using QIIME software 2023.5. Then, reads with chimera were detected and removed using VSEARCH 2.28.1. Clean reads were subjected to primer sequences removal and clustering to generate operational taxonomic units (OTUs) using Abundance-based Greedy Clustering algorithm with 97% similarity cutoff. The representative read of each OTU was selected using QIIME package. All representative reads were annotated and blasted against Silva database (Version 132) using RDP classifier (confidence threshold was 70%) [20].

Alpha diversity indices were calculated to evaluate the diversity of bacterial community structure. The Chao1 index was used to estimate community richness, while the Shannon diversity index and the Simpson diversity index were employed to assess community diversity. All the aforementioned indices were computed using Mothur 1.43.0, with the input dataset undergoing neither subsampling nor screening, given the adequate sequence depth. Beta diversity was analyzed to demonstrate the similarities or disparities among the artificial samples using Principal Components Analysis (PCA), with the application of Aitchison distances for metrics calculations.

## 3. Results

### 3.1. Physicochemical Properties and Bacterial Communitry Structure of the Collected Groundwater

The physiochemical properties of the collected groundwater used in the artificial samples are shown in Table 2. The groundwater was mildly acidic and in a reducing state, as evidenced by a pH of 5.89 and an ORP of −86 mV. The DO, NO_3_^−^, and SO_4_^2−^ concentrations, serving as electron acceptors in microbial-mediated redox reactions, were found to be low. The primary contaminants were benzene and toluene, with benzene at 209.00 mg/L and toluene at 5.18 mg/L. Other contaminants, predominantly comprising ethylbenzene and xylene, were present at relatively low levels and were thus disregarded in the batch artificial sample study. Consequently, the limited availability of electron acceptors, the negative ORP, and the high contaminant concentrations collectively suggest that the environment is conducive to anaerobic microbial degradation of contaminants in the groundwater sample [6].

**Figure 2:**
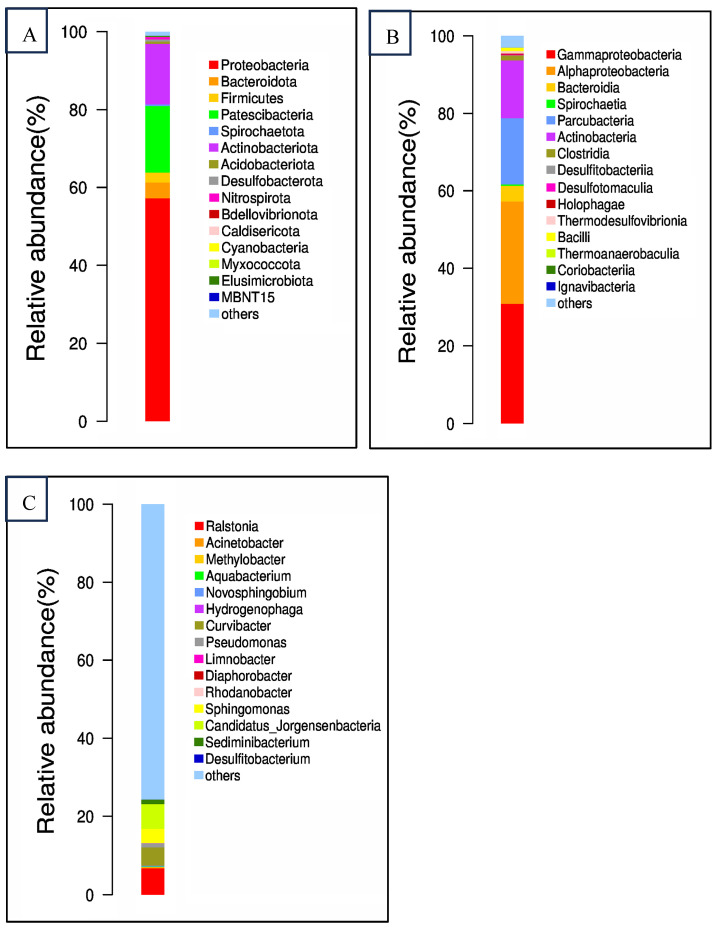
Bacterial communities of the groundwater sample at phylum level (**A**), class level (**B**) and genus level (**C**).

The richness of bacterial communities in the collected groundwater, as measured by Chao1 values, is 1229.74. The Shannon and Simpson index values in the collected groundwater are 5.29 and 0.9391, respectively. Figure 2 shows the bacterial community structure of the groundwater at phylum, class, and genus levels. 

Prior to sulfate addition, the dominant phyla detected in the artificial samples were Proteobacteria, Bacteroidota, Firmicutes, Patescibacteria, and Actinobacteriota. Proteobacteria, the most abundant phylum, encompasses a diverse array of facultative or obligate anaerobic microorganisms frequently encountered in wastewater cultures and groundwater sediments [21]. These organisms have been demonstrated to possess the ability to degrade BTEX (benzene, toluene, ethylbenzene, and xylenes) compounds. Bacteroidota, typically recognized as heterotrophic aerobic and nitrifying bacteria, are capable of degrading a wide range of organic substances [22]. Firmicutes and Actinobacteria, increasingly identified in various phylogenetic lineages, are known to encompass manganese-oxidizing bacteria (MnOB) [3] and play significant roles in nitrate reduction processes. Patescibacteria, ubiquitously present in groundwater, sediments, and lakes, harbor a repertoire of organic-active enzymes that facilitate the degradation of complex organic matter into simpler molecules, as evidenced by previous research [22].

At the class level, the bacterial community in the artificial samples prior to sulfate addition was primarily composed of Gammaproteobacteria, Alphaproteobacteria, Bacteroidia, Parcubacteria, and Actinobacteria. It is noteworthy that Gammaproteobacteria and Alphaproteobacteria have been documented as the dominant classes in BTEX-contaminated groundwater ecosystems [3]. Bacteroidia, on the other hand, are believed to play a crucial role in the decomposition of complex molecules into simpler compounds, particularly in the utilization of nitrogenous substances under strictly anaerobic conditions [21].

At the genus level, *Ralstonia*, *Curvibacter*, *Sphingomonas*, and *Candidatus* genera were among the most prevalent at the genus level. Ralstonia species have been isolated from oil-contaminated soil samples [23], demonstrating their potential role in hydrocarbon degradation. *Sphingomonas*, being a strictly aerobic bacterium, is known for its capability to degrade macromolecular organic contaminants, particularly polycyclic or monocyclic aromatic compounds. It thrives in environments with contaminants, showing higher population densities when contaminants are present. *Curvibacter*, on the other hand, has been identified to degrade BTEX (Benzene, Toluene, Ethylbenzene, and Xylenes) under hypoxic conditions, which underscores its significance in anaerobic or low-oxygen environments. Each of these genera contributes to the natural attenuation of contaminants in groundwater systems, and their populations can shift in response to sulfate addition and varying contaminant concentrations, highlighting their adaptability to different environmental conditions.

### 3.2. Biodegradation of Benzene and Toluene in the Artificial Samples

**Figure 3:**
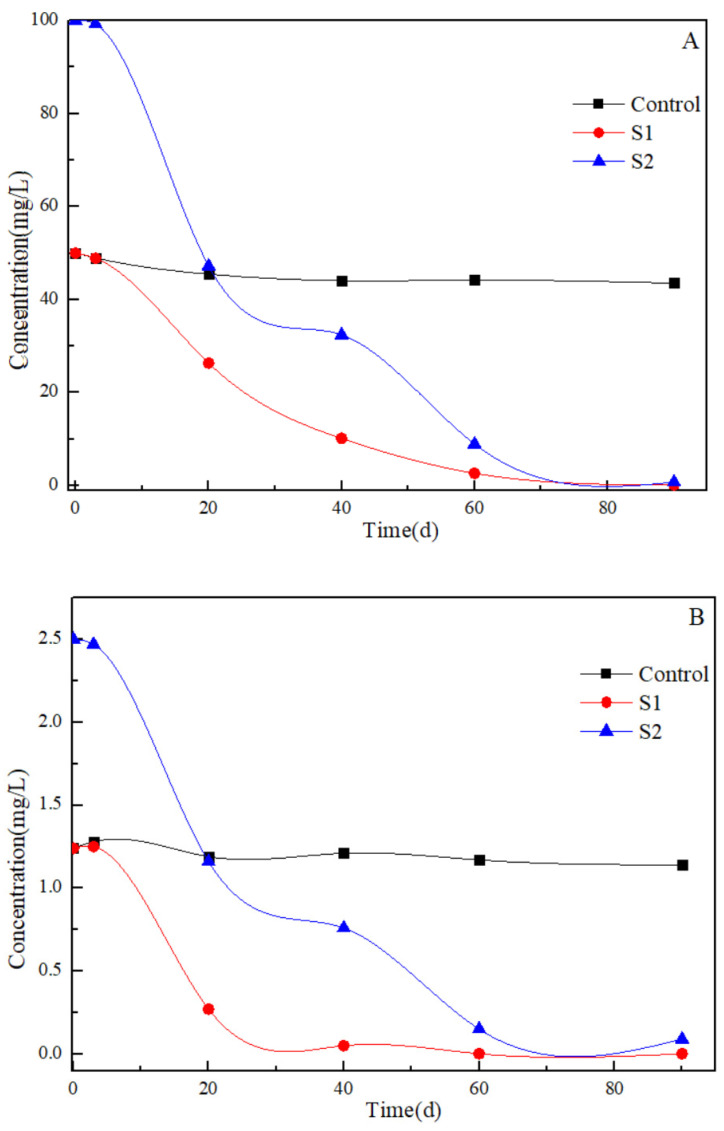
Changes in benzene (**A**) and toluene (**B**) concentration during the 90 days period.

The efficacies of concurrent benzene and toluene elimination under sulfate-reducing conditions in the artificial samples were assessed. Figure 3 depicts the changes of concentrations of benzene and toluene over the 90-daystudy. Remarkably, approximately 99% of benzene was removed in both S1 and S2 mcirocosms within 90 days. Similarly, approximately 96% of toluene was eliminated in both S1 and S2 artificial samples over the same period. In contrast, the concentrations of benzene and toluene in the Control artificial sample remained constant due to microbial inactivation. The degradation kinetics of benzene and toluene in S1 and S2 generally follow a first-order reaction pattern. In S1, the first-order degradation rate constants are as follows: 0.06744 d^−1^ for benzene and 0.08118 d^−1^ for toluene. In S2 artificial sample, the corresponding first-order degradation rate constants are 0.05252 d^−1^ for benzene and 0.03955 d^−1^ for toluene. Notably, the degradation rates of benzene and toluene in S1 and S2 artificial samples were influenced by the concentrations of these pollutants. 

### 3.3. Changes of Geochemcial Parameters in the Artificial Samples

**Figure 4:**
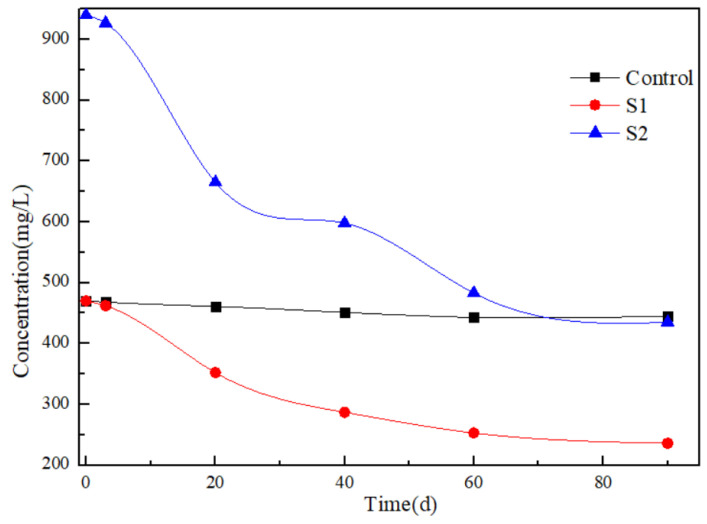
Changes in sulfate concentration during the 90 days period.

Figure 4 illustrates the variations in sulfate concentration within the artificial samples. Based on the theoretical sulfate requirement for benzene biodegradation, every gram of benzene degradation necessitates 4.7 g of sulfate. In the S1 artificial sample, the sulfate concentration dwindled from an initial 470 mg/L to 352 mg/L and further to 287 mg/L after 20 and 40 days, respectively. Concurrently, in the S2 artificial sample, the sulfate concentration descended from 940 mg/L to 665 mg/L and then to 598 mg/L following 20 and 40 days, respectively. Overall, the decline in sulfate concentration approximately mirrors a 4.7-fold reduction in benzene concentration. The observed sulfate concentration alterations align with the predicted sulfate consumption. As expected, the sulfate concentration in the Control artificial sample remained unaltered throughout the experiment. It is noteworthy that reported ratios between sulfate reduction and contaminant degradation were 3.51 and 4.33 for benzene and toluene, respectively, which were strikingly close to their theoretical counterparts [24]. This congruity substantiates the significant role of sulfate reduction in mediating the biodegradation of benzene and toluene in these artificial samples.

Thus, the observed anaerobic biodegradation of organic compounds in the artificial samples can be attributed to the sulfate-reducing process. The introduction of additional sulfate into the S1 and S2 artificial samples facilitated sulfate reduction as the predominant mechanism for benzene and toluene degradation. Essentially, microorganisms within these artificial samples utilized sulfate as the electron acceptor and benzene as the electron donor, thereby facilitating the degradation of benzene. This finding confirms the pivotal role of sulfate-reducing bacteria in mediating the anaerobic breakdown of benzene and toluene under sulfate-rich conditions.

Figure S1 presents the fluctuations in pH and ORP values throughout the artificial sample study. The pH values in S1 and S2 artificial samples were slightly higher than that of the Control group, but still remained within the neutral range, which was favorable for microbial activity. The Control artificial sample’s ORP remained relatively stable, fluctuating around −50 mV. In contrast, the ORP values in S1 and S2 artificial samples experienced a rapid decrease after 3 days. Subsequently, S1 artificial sample stabilized at around −140 mV, while S2 artificial sample maintained a level of approximately −120 mV. The significant decrease in ORP values observed in S1 and S2 after 3 days indicates a shift in the redox state, likely due to increased microbial activity in the availability of electron donors and acceptors. The stabilization of ORP at lower values in these artificial samples suggests that the system has adapted to a new set of conditions, potentially indicating a more reducing environment. 

### 3.4. Changes of Bacterial Community Structures in the Artificial Samples

As demonstrated earlier, the introduction of sulfate significantly promoted sulfate reduction as the primary pathway for benzene and toluene biodegradation in the S1 and S2 artificial samples. This observation strongly suggests the presence of microorganisms within the artificial sample environment possessing sulfate-reducing capabilities, which actively participated in the degradation of the target contaminants. These sulfate-reducing bacteria likely utilized sulfate as an electron acceptor and benzene/toluene as electron donors, driving the anaerobic degradation process observed in the study.

Table 3 displays the richness and alpha diversity estimators of the bacterial communities present in the artificial samples. In both S1 and S2 artificial samples, the richness of bacterial communities, as measured by Chao1 values, initially decreased and subsequently increased over time. The Shannon and Simpson index values, which are proxies for community diversity, demonstrated a similar trend for both artificial samples. This pattern is likely a consequence of the addition of sulfate, which altered the groundwater environment and fostered the proliferation of sulfate-reducing bacteria. As the sulfate reducers progressively became the dominant bacterial species following sulfate supplementation [4], a temporary decrease in bacterial diversity and richness was observed at day 40, however, by day 90 bacterial diversity and richness exhibited a substantial increase. Given that the contaminants have become negligible at this stage, they no longer exerted a strong selective pressure on the bacterial community, allowing for the resurgence of bacterial diversity in the later stages of the incubation under sulfate-reducing conditions.

Furthermore, throughout the entire artificial sample study, the Chao 1 value for the S1 artificial sample consistently surpassed that of the S2 artificial sample. A parallel trend was observed in the Shannon and Simpson index values. This indicated a correlation between bacterial richness and diversity and the contaminant concentration, where higher level of contaminants exerted more toxic effects on bacterial growth. This phenomenon was corroborated in the literature through comparisons of results at multiple sampling points along groundwater flow paths [25].

**Figure 5:**
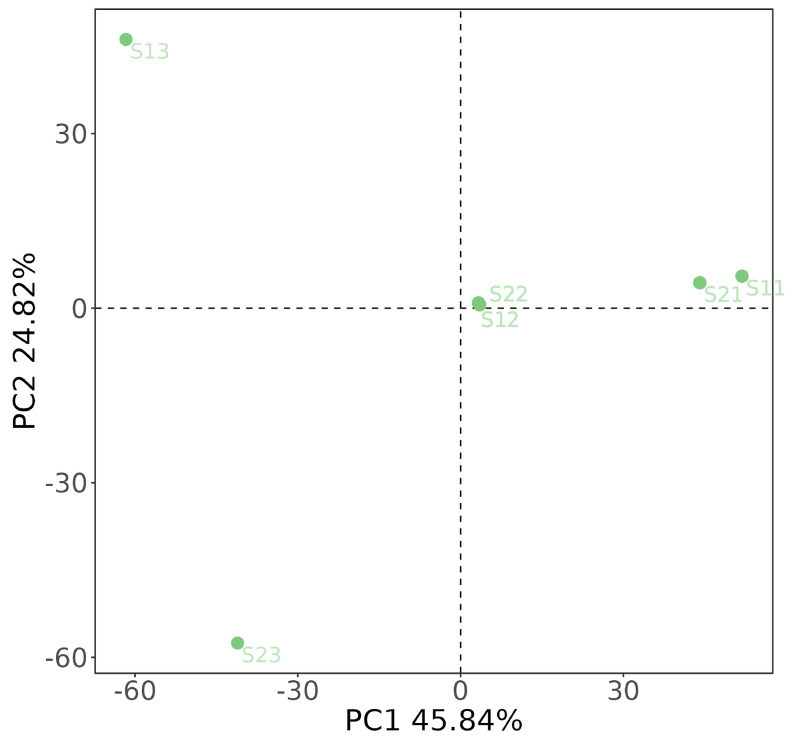
Two-dimensional PCA of the S1 and S2 artificial samples using the Aitchison distances.

To illustrate the relationship between microbial structures in various artificial samples, two-dimensional PCA plots were presented in Figure 5. It is well understood that the distances between samples are crucial for analysis within a PCA plot. Typically, samples that cluster closely together indicate a higher degree of similarity in their microbial community composition, while those that are more widely spaced suggest greater disparities in community structure. Utilizing PCA with Aitchison distances, the S1 and S2 artificial samples, collected during the same reaction period at 3 d and 40 d, clustered closely together, indicating a high level of similarity in their microbial community structures. In contrast, artificial samples S13 and S23, taken after 90 days of reaction, were notably distant from each other on the PCA plot, highlighting a substantial difference in their respective microbial communities. Conversely, samples from the same group but obtained at disparate reaction stages appeared more distantly located on the PCA plot, reflecting a marked dissimilarity in their microbial compositions as time progressed. These alterations in microbial community structures correlate with fluctuations in contaminant concentration levels.

**Figure 6:**
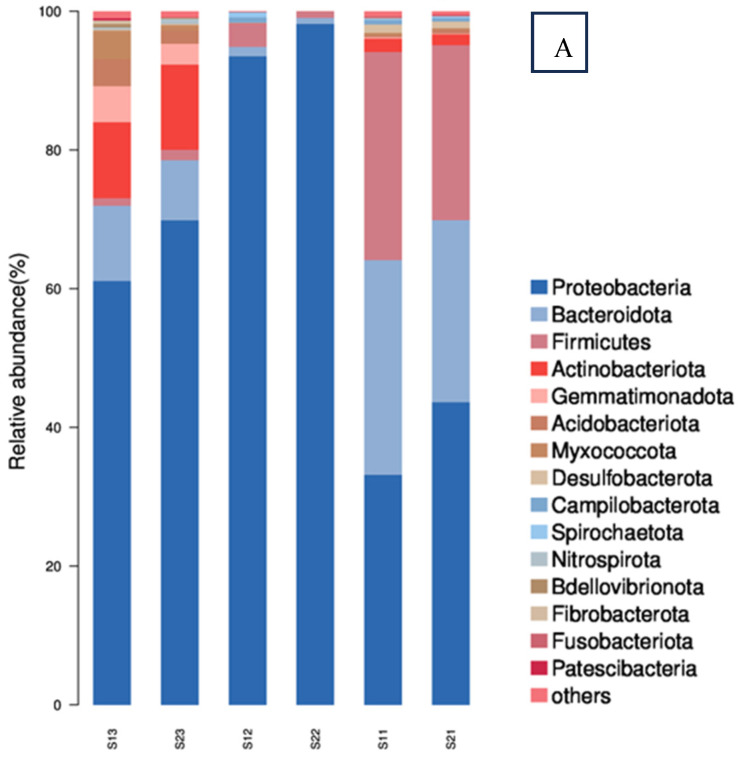

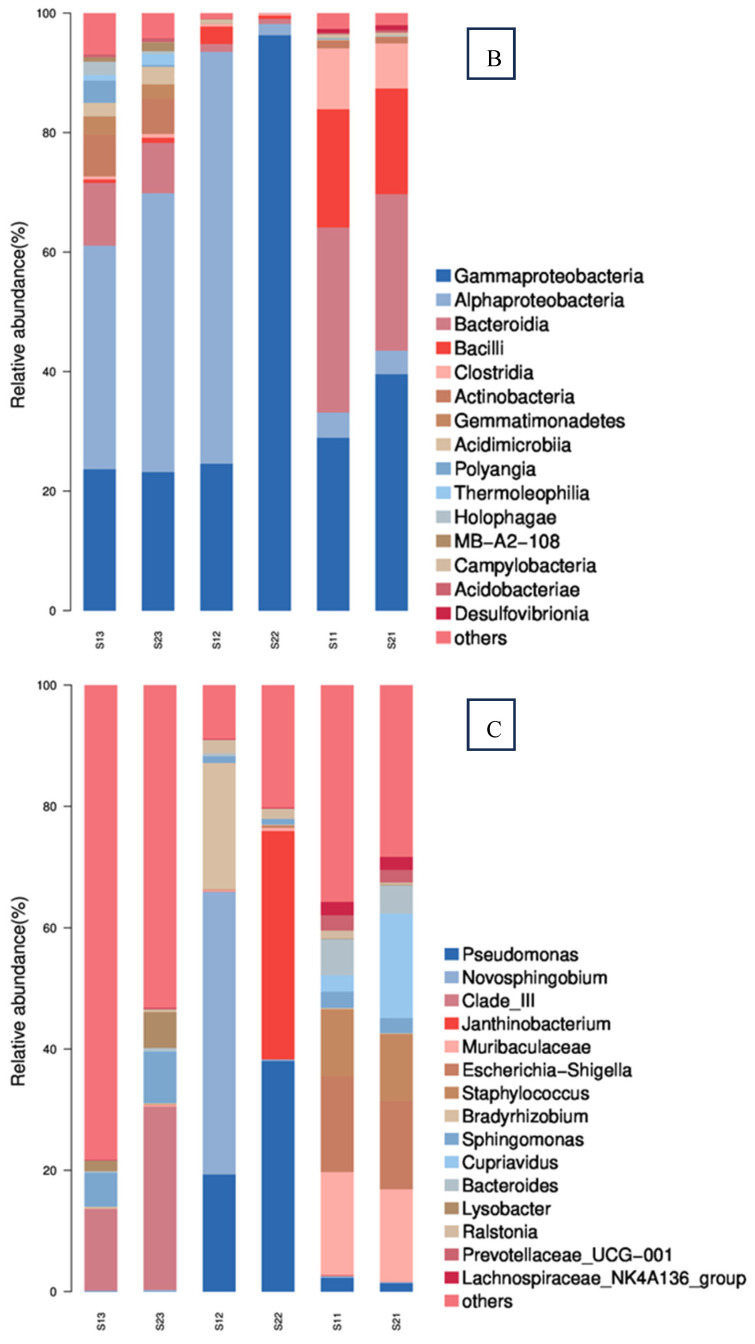
Bacterial communities of the treated groundwater samples at phylum (**A**), class (**B**), and genus (**C**) levels (S11 represents S1 at 3 days; S12 represents S1 at 40 days; S13 represents S1 at 90 days; S21 represents S2 at 3 days; S22 represents S2 at 40 days; S23 represents S2 at 90 days).

Figure 6 shows the comparisons of bacterial community structures in different samples. Following the addition of sulfate at day 3, the dominant phyla detected shifted to Proteobacteria, Bacteroidota, and Firmicutes, with a marked decrease in the relative abundance of other phyla. At day 40, the proportion of Proteobacteria reached its peak, coinciding with a substantial decline in contaminant concentrations, although still sufficient to exert a selective pressure on the microbial population. By day 90, with contaminants present at negligible levels, bacterial diversity rebounded significantly. The fluctuations in phylum-level composition parallel the trends observed in the Shannon and Simpson diversity indices, indicating that the dominance of Proteobacteria, Bacteroidota, and Firmicutes in the groundwater artificial samples during this period was accompanied by a substantial reduction in bacterial diversity.

At the class level, at day 3, following sulfate addition, Gammaproteobacteria, Alphaproteobacteria, Bacteroidia, and Bacilli continued to dominate the bacterial community at the class level, displaying a resemblance to the community structure in the absence of sulfate. At day 40, the bacterial community was predominantly comprised of Gammaproteobacteria and Alphaproteobacteria, whereas at day 90, Gammaproteobacteria, Alphaproteobacteria, and Bacteroidia regained their dominance. 

At the genus level, at day 3 post-sulfate addition, *Pseudomonas*, *Staphylococcus*, *Novosphingobium*, and *Bradyrhizobium* began to flourish and emerged as the dominant genera in the bacterial community. At day 40, *Pseudomonas*, *Janthinobacterium*, and *Novosphingobium* took precedence. The presence of these genera is consistent with their known involvement in aromatic hydrocarbon degradation. *Pseudomonas*, in particular, plays a pivotal role in benzene and naphthalene biodegradation under sulfate-reducing conditions. The bacteria detected in both artificial samples at day 40 are likely anaerobic, contributing to benzene and toluene degradation under anaerobic circumstances. Notably, *Pseudomonas* and *Janthinobacterium* were more dominant in the heavily contaminated S2 groundwater, while *Pseudomonas* and *Novosphingobium* were more prominent in the less contaminated S1 groundwater. By day 90, *Clade* and *Staphylococcus* topped the list of dominant genera, with a notably higher diversity compared to day 40. These findings highlight the significant shifts in the bacterial community structure over time. The bacterial community in the sulfate-treated groundwater samples differed significantly from that of the pre-sulfate addition samples. The results reveal that the bacteria present in the artificial samples at day 3, 40, and 90 did not maintain a consistent relationship, signifying that the contaminants and sulfate supplementation led to substantial changes in bacterial diversity within the artificial samples. This transformation could be due to the altered physical and chemical conditions in the artificial samples brought about by the sulfate addition and changing contaminant levels.

At the same time intervals, it’s evident that the bacterial domestication, or adaptation to specific conditions in the S2 artificial sample was more pronounced compared to the S1 mcirocosm. This difference can be attributed to the higher contaminant concentration in the S2 artificial sample. Excessive contaminant levels can exert toxic effects on bacterial growth, which can lead to selection pressures that favor the survival and proliferation of certain bacterial populations better adapted to tolerate or degrade the contaminants [26]. Consequently, the more contaminated S2 artificial sample experienced a more dramatic shift in its bacterial community composition, reflecting a stronger domestication process as it adjusted to the sulfate-reducing conditions and the presence of high contaminant loads.

The original groundwater sample, which did not receive sulfate addition, exhibited a more diverse bacterial community compared to the samples at day 40 post-sulfate treatment. Conversely, the sulfate-amended samples harbored predominantly anaerobic sulfate-reducing bacteria, such as *Pseudomonas*, *Janthinobacterium*, and *Novosphingobium*, which was in line with the ample availability of sulfate as an electron acceptor in these artificial samples. Moreover, the temporal changes in bacterial community richness and diversity closely mirrored the fluctuation in contaminant concentrations. Based on the results, several bacterial genera were identified as benzene/toluene-degrading bacteria under sulfate-reducing conditions. These include *Pseudomonas*, *Janthinobacterium*, *Novosphingobium*, *Staphylococcus*, and *Bradyrhizobium*. These findings underscore the critical role played by these bacteria in the anaerobic degradation of benzene in the presence of sulfate. 

Understanding the dynamics of these microbial communities provides a scientific foundation for optimizing bioremediation strategies in the following ways.

1. Enhancing activities: By adjusting environmental parameters such as pH, ORP, and sulfate availability for the growth and metabolic activity of these bacteria, it is possible to enhance their degradation capabilities.
2. Targeted introduction: Knowing these bacteria are most effective at degrading benzene can guide the targeted introduction of these species into contaminated environments. This approach can be more effective than a general inoculation with an undefined microbial mixture.
3. Monitoring and adjustment: Regular monitoring of the microbial community structure can help assess the progress of bioremediation and allow for timely adjustments to the strategy.

In conclusion, the study of these specific bacterial communities and their roles in the anaerobic degradation of benzene provides valuable information that can be used to improve the effectiveness of bioremediation strategies. This knowledge not only contributes to the remediation of contaminated sites but also enhances our understanding of the complex microbial interactions that occur in the environment.

## 4. Conclusions

This study investigated the shifts in the bacterial community structure during the biodegradation of benzene and toluene under sulfate-reducing conditions. In both S1 (benzene 50 mg/L, toluene 1.24 mg/L, sulfate 470 mg/L) and S2 (benzene 100 mg/L, toluene 2.5 mg/L, sulfate 940 mg/L) artificial samples, approximately 99% benzene and 96% toluene were removed from the water over a 90-day period. The contaminant concentration has a profound impact on the abundance and diversity of the bacterial communities. As the contaminant concentration decreased, bacterial abundance and diversity tended to increase, logically consistent with the notion that excessively high contaminant levels can exert toxic effects on bacterial growth. Under sulfate-reducing conditions, key players in benzene/toluene degradation were identified as *Pseudomonas*, *Janthinobacterium*, *Novosphingobium*, *Staphylococcus*, and *Bradyrhizobium*. Specifically, *Pseudomonas* and *Janthinobacterium* were found to dominate in the more heavily contaminated groundwater samples, while *Pseudomonas* and *Novosphingobium* were more prevalent in the less contaminated samples. The biological remediation under sulfate reduction processes provides a promising, efficient, and cost-effective strategy for remediating BTEX-contaminated groundwater. By harnessing the metabolic capabilities of sulfate-reducing bacteria and understanding their responses to varying contaminant concentrations, this approach can enhance the effectiveness of groundwater cleanup efforts.

## Figures and Tables

**Table 1:** Components of three artificial samples.

Artificial Sample	Components
Control	contaminated groundwater containing benzene (50 mg/L) and toluene (1.24 mg/L) + 250 mg/L HgCl_2_+ sulfate (470 mg/L) + yeast extract (50 mg/L)
S1	contaminated groundwater containing benzene (50 mg/L) and toluene (1.24 mg/L) + sulfate (470 mg/L) + yeast extract (50 mg/L)
S2	contaminated groundwater containing benzene (100 mg/L) and toluene (2.5 mg/L) + sulfate (940 mg/L) + yeast extract (50 mg/L)

**Table 2:** Physiochemical properties of the collected groundwaters used in the artificial samples.

pH	ORP (mV)	DO (mg/L)	NO_3_^−^ (mg/L)	SO_4_^2−^ (mg/L)	Fe^2+^ (mg/L)	Mn^2+^ (mg/L)	Benzene (mg/L)	Toluene (mg/L)	Ethylbenzene (mg/L)	m, p-Xylene (mg/L)	o-Xylene (mg/L)
5.89	−86	0.75	0.797	2.82	8.84	2.65	209.000	5.180	0.460	0.110	0.188

**Table 3:** The richness, diversity estimators and alpha diversity estimators of bacterial community in the S1 and S2 artificial samples.

Samples	Chao1	Shannon	Simpson	Observed Species	Goods Coverage	PD-Whole Tree
S11 (3 d)	581.33	7.18	0.9674	580.50	0.9999	50.24
S12 (40 d)	133.13	2.56	0.7016	132.60	0.9999	17.44
S13 (90 d)	1429.26	8.81	0.9780	1422.90	0.9994	101.80
S21 (3 d)	513.53	6.41	0.9430	512.70	0.9999	46.19
S22 (40 d)	109.72	2.08	0.6815	108.50	0.9999	15.82
S23 (90 d)	1072.56	7.18	0.9075	1068.00	0.9996	77.54

## Data Availability

The original data presented in the study are included in the article; further inquiries can be directed to the corresponding author.

## Supplementary Materials

The following supporting information can be downloaded at: https://www.mdpi.com/article/10.3390/toxics12060423/s1, Table S1. Polymerase chain reaction (PCR) reaction system and reaction conditions. Figure S1. Changes of pH and ORP during the 90 days period.
