# Supplementary material for: Shift in Bacterial Community Structure in the Biodegradation of Benzene and Toluene under Sulfate-Reducing Condition
Source: Toxics. 2024 Jun 10;12(6):423. doi: 10.3390/toxics12060423 (PMC11209115; doi:10.3390/toxics12060423)
Supplement: Supplementary file 1 [file toxics-12-00423-s001.zip › toxics-3023274-supplementary.pdf]

Table S1. Polymerase chain reaction (PCR) reaction system and reaction conditions.

PCR reaction system		Reaction condition		
2×Gflex PCR Buffer	15 μL	94 °C	5 min	
DNA	≥1 μL(50 ng)	94 °C	30 s	26 cycles
5 pmol/μl primer F	1 μL	56 °C	30 s	
5 pmol/μl primer R	1 μL	72 °C	20 s	
Tks Gflex DNA Polymerase (1.25 U/μL)	0.6 μL	72 °C	5 min	
H_2O	Added to 30 μL	4 °C	hold	
Total	30 μL			

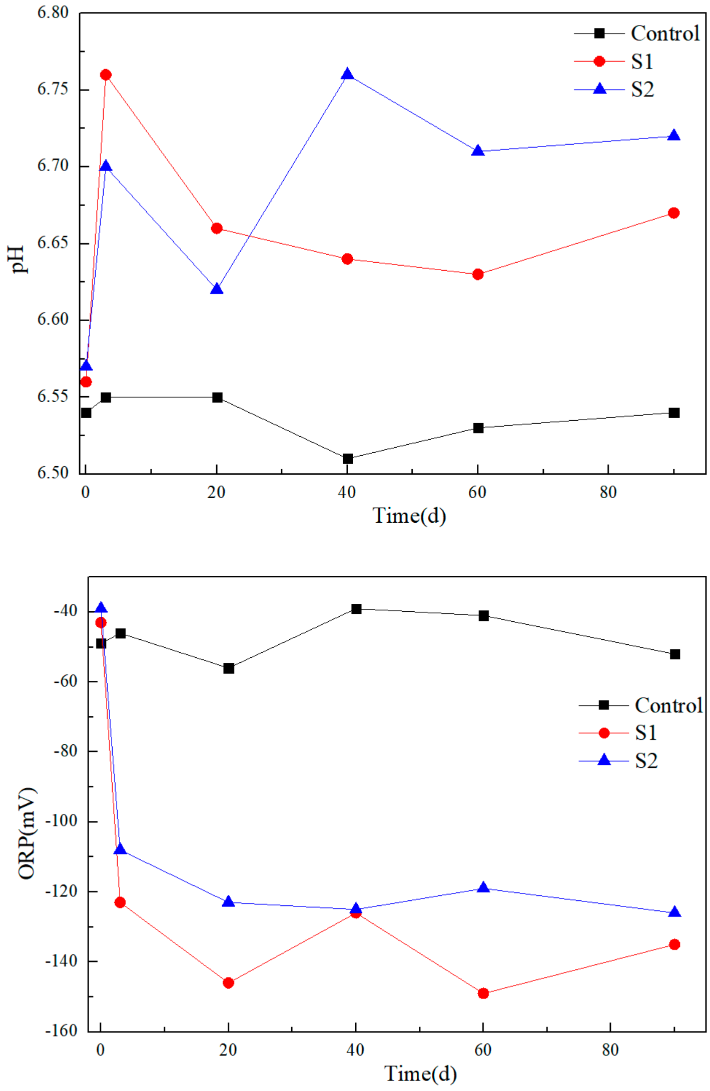

Figure S1. Changes of pH and ORP during the 90 days period.
